# Nanoscopic biodosimetry using plasmid DNA in radiotherapy with metallic nanoparticles

**DOI:** 10.1002/acm2.13879

**Published:** 2022-12-22

**Authors:** Elham Mansouri, Asghar Mesbahi, Mohammad Saied Hejazi, Soheila Montazersaheb, Vahideh Tarhriz, Tohid Ghasemnejad, Mojtaba Zarei

**Affiliations:** ^1^ Drug Applied Research Center Tabriz University of Medical Sciences Tabriz Iran; ^2^ Molecular Medicine Research Center Institute of Biomedicine Tabriz University of Medical Sciences Tabriz Iran; ^3^ Medical Physics Department Medical School Tabriz University of Medical Sciences Tabriz Iran

**Keywords:** biodosimetry, DNA plasmid, nanoparticle, radiation therapy

## Abstract

Nanoscopic lesions (complex damages), are the most lethal lesions for the cells. As nanoparticles have become increasingly popular in radiation therapy and the importance of analyzing nanoscopic dose enhancement has increased, a reliable tool for nanodosimetry has become indispensable. In this regard, the DNA plasmid is a widely used tool as a nanodosimetry probe in radiobiology and nano‐radiosensitization studies. This approach is helpful for unraveling the radiosensitization role of nanoparticles in terms of physical and physicochemical effects and for quantifying radiation‐induced biological damage. This review discusses the potential of using plasmid DNA assays for assessing the relative effects of nano‐radiosensitizers, which can provide a theoretical basis for the development of nanoscopic biodosimetry and nanoparticle‐based radiotherapy.

## 1 INTRODUCTION

The accuracy of radiation dose delivery and treatment predictability are the cornerstones of radiation therapy. Furthermore, radiation dosimetry is crucial for the maintenance, and management of accurate and precise radiotherapy for cancer patients and safety of radiotherapy staff. Generally, a better understanding of the radiation damage mechanisms will lead to more accurate predictions of radiation risks. Radiation absorbed dose alone is not sufficient in predicting the biological effects of ionizing radiation in tissues.^1^ Notably, dosimetry in conventional methods does not take into account the biological effects of radiation or the sensitivity of human tissues and organs to the range of ionizing radiation.^2^ While, in radiation biodosimetry, the dose is estimated by monitoring changes in physiological, chemical, or biological markers after irradiation with ionizing radiation.^2^ In other words, radiation biodosimetry refers to the process by which the properties of biological molecules are quantitatively altered in response to ionizing radiation. Several biodosimetry methods and procedures such as enzyme‐linked immunosorbent assay (ELISA), nanometric sensors, and electrochemical sensors have been proposed to determine the changes of biomarkers.^3^ A DNA damage caused by radiation is the first step of radiation damage to a gene or a cell. In fact, radiation‐induced DNA damage is initiated by the inelastic interactions between DNA molecules and their surroundings. The amount of energy deposition can be measured in a medium in a macroscopic (millimetric) scale using physical dosimeters, such as ionization chambers, scintillators, thermoluminescence dosimeters, and film badges. The impact of nano‐radiosensitizers cannot be explained in elevated macroscopic energy deposition. Instead, local disturbances in nanoscale must be considered to describe the enhancement of radiation effects. In studies of radio‐enhancers, such as high‐Z NPs, DNA plasmids are widely used tools as a nanodosimetry probe to investigate radiation‐induced damage.^4^, ^5^, ^6^, ^7^, ^8^, ^9^, ^10^, ^11^, ^12^, ^13^, ^14^, ^15^, ^16^, ^17^, ^18^, ^19^, ^20^ They have the advantage of being small DNA molecules, providing precise control over the scavenging environment and irradiation settings, as well as being easy to quantify by electrophoresis.^21^ Furthermore, DNA plasmids are convenient for unraveling the physical/physicochemical radiosensitization mechanisms of nanoparticles (NPs) and quantifying the biological damages of radiation. This review aims at shedding light on various aspects of using different DNA plasmids as a molecular biodosimeter in nano‐radiosensitization studies.

## 2 THE DNA PLASMID AS A BIODOSIMETER FOR NANO‐RADIOSENSITIZATION STUDIES

**FIGURE 1:**
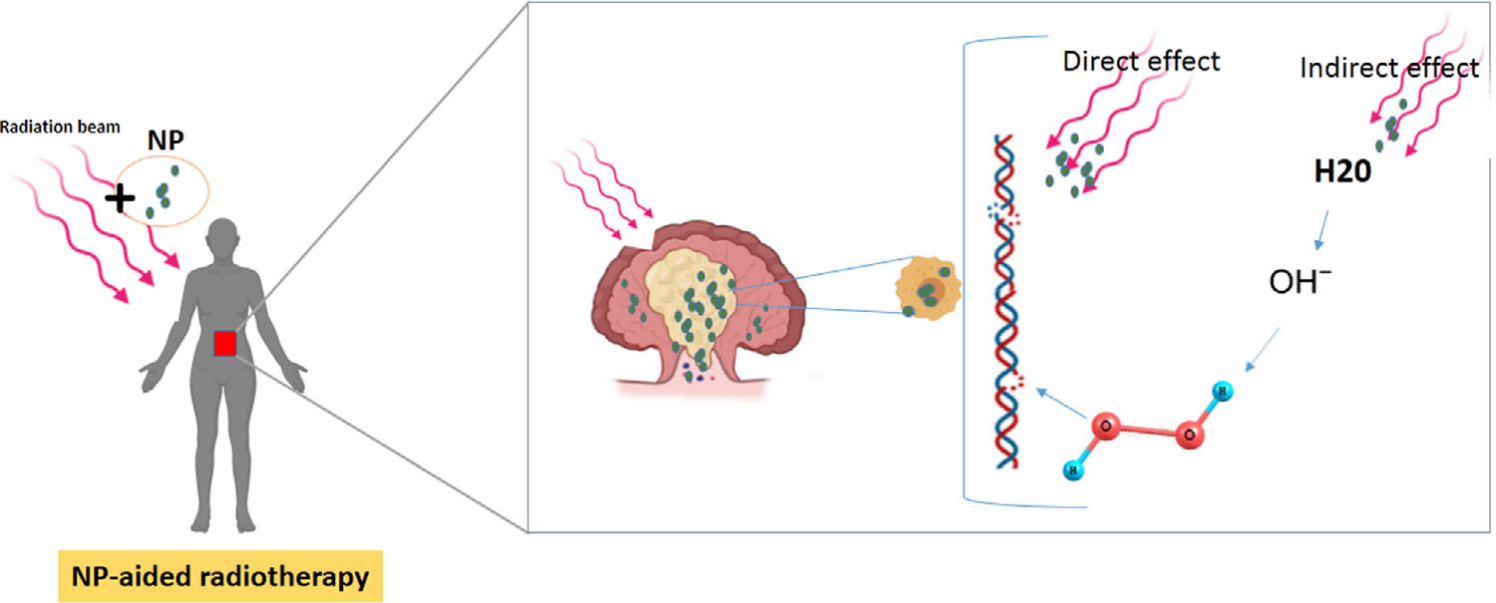
Schematic description about NP‐aided radiotherapy

Tumor‐specific NPs have improved radiation therapy outcomes by increasing toxicity for tumors and inducing less toxicity for normal tissues.^22^ This function is performed by enhancing ionizing energy deposition from the nanometer to the micrometer range at tumor cells (Figure 1). Radiation therapy using NPs of high atomic number (Z), such as noble metals and lanthanides, offers a promising new treatment option for cancers.^23^ The influence of NPs in increasing efficiency of radiation therapy is measured by the dose enhancement factor (DEF) and enhancement factor (EF). Here, the EF is defined as the ratio of the DNA damages in the presence and lack of NPs, and DEF refers to the ratio of the delivered dose in the presence and absence of NPs. There has been considerable work on the radiosensitizing efficacy of NPs, including mathematical,^24^, ^25^ in silico,^26^, ^27^, ^28^, ^29^, ^30^, ^31^, ^32^, ^33^, ^34^, ^35^ and in various biological systems including molecular solutions,^5^, ^36^, ^37^, ^38^ cell‐based models^39^, ^40^, ^41^, ^42^, ^43^, ^44^ and in vivo.^45^, ^46^ Ionizing radiation causes a variety of direct and indirect damage to DNA, including isolated (single‐strand break (SSB) and single base damages) and clustered/complex lesions (multiple DSBs and/or closely spaced (within 10–20 base pairs) non‐DSB (double strand break) lesions, such as oxidized bases and abasic sites) that affect genome integrity and DNA biochemistry.^21^, ^47^, ^48^ The damages can be induced even by a single radiation track through a cell. An experiment conducted by Porcel et al.^49^ confirms that the addition of NPs can amplifies molecular damages, particularly, the DNA breaks of nanometer size.

Although it is difficult to directly probe the nanoscopic energy deposition and energy distribution, it is possible to indirectly measure it by using biological samples such as DNA molecules in a controlled environment. As it is believed that ionizing radiation primarily affects cells and tissues by causing DNA damage, thereby DNA plasmid is a beneficial tool for indirectly studying radiobiological effects of ionizing radiations without many of the complications associated with cellular environments. Conventionally, plasmid DNA solvation used to represent a simplified model of the cell nucleus.^50^ DNA plasmids (closed double‐stranded DNA loops compromising several thousand base pairs) are commonly used as a surrogate to study DNA damages such as SSBs and DSBs to quantify the effects of ionizing radiations combined with nanoparticles (NPs) in biologically relevant conditions. Several studies experimentally analyzed the formation of plasmid DNA‐DSB and ‐SSB as dosimetry standards. DSB indicate the nanometer‐scale damage which belongs to the most complex and severe types of DNA damages since it directly affects genome integrity and cellular survival.^51^ While, SSBs are less severe to genome integrity because an intact template strand is still available, ensuring complementarity‐aided, error‐free repair of the lesion.

Several radiosensitization studies have used DNA plasmids as a nanodosimeter to study the parameters governing the radiosensitization potential of NPs in NP‐aided radiation therapy (Table 1). Plasmid DNA is selectively bound and purified from RNA, proteins, and other cellular contaminants. Relying on this concept, plasmid‐DNA:NP molar ratio, the physicochemical properties of the NP (size, shape, coating, functionalization, etc.), radiation conditions (energy, dose rate, dose, linear energy transfer (LET)), the plasmid type, and sample environment are governing parameters in efficacy of plasmid DNA as a nanodosimetry probe in radiosensitization studies which will be discussed in the following sections.

**TABLE 1:** Summary of in‐vitro studies using plasmid DNA as biodosimetry probe in nano‐radiosensitization evaluations

Author/study	Plasmid/sample type	NP	Plasmid/NP molar ratio	Irradiation conditions	Main findings
Butterworth et al.^6^	Pcdna3.1 5400 bp Aqueous environment	AuNP 5 nm	–	tube voltage 160 KVp, 14.3 Gy/min	Plasmid damage yields is highly dependent on scavenging capacity of preparation buffers.
Kiril et al.^38^	pBR322 4361 bp Aqueous environment	AuNP 26 nm	1:3–1:60 (1.5–0.075‐particles per one DNA molecule)	5 Gy tube voltage: 200 kVp 0.2 Gy/min	Enhancement factor increased from 1 to 1.4 by increasing the NP concentration from 0.6 to 2.4 mg/mL.
Kiril et al.^38^	pBR322 4361 bp Aqueous environment	AuNP 26 nm	1:3 to 1:60 (1.5–0.075‐particles per one DNA molecule)	5 Gy tube voltage: 200 kVp 0.2 Gy/min, 0.41 Gy/min, 0.85 Gy/min, 1.36 Gy/min, and 2.1 Gy/min	The change in the dose rate did not affect DNA damage in the absence of AuNPs. In contrast, in the presence of AuNPs, a noticeable change in the magnitude of this effect was observed. The Highest Radiosensitization by 26 nm AuNPs was achieved at the lowest dose rate.
Kiril et al.^38^	pBR322 4361 bp Aqueous environment	AuNP 26 nm	1:3 1.5 NP per one DNA molecule)	5 Gy tube voltage: 100 150 200 kVp0.2 Gy/min	The increase in the voltage on the X‐ray tube significantly enhances the AuNPs radio‐sensitizing effect
Kiril et al.^38^	pBR322 4361 bp Aqueous environment	AuNP 12,15, 21 and 26 nm	15.3 (12 nm), 7.8 (15 nm), 2.8 (21 nm), and 1.5 (26 nm) particles per one DNA molecule.	5 Gy tube voltage: 100 150 200 kVp0.2 Gy/min	Enhancement exhibited a linear dependence of enhancement. The greatest radiosensitization corresponded to the largest AuNP size. with AuNP diameter
McMahon et al.^5^	pcDNA3.1 Aqueous environment	AuNP 11.9 nm	4:1	11.8–60 keV 0–50 Gy	Presence of AuNPs in plasmid DNA samples leads to increase in the rate of single‐strand breaks by 25%.
McMahon et al.^5^	pUC18 and 2686 bp Aqueous environment	AuNP 37.5 nm	4:1	14.8 keV 24.4 keV 29.8 keV 42.4 keV 49 keV 70 keV	Dose enhancement that occurs by using AuNPs with high‐energy photons is predicted to be much smaller than that observed with lower energy photons.
Zheng et al.^16^	pGEM‐3Zf 3197 bp Dry films	AuNP 1 to 12 nm	1:30, 2:5, 1:1, 4:3 and 2:1.	electron60 keV	The X‐ray irradiation would induce increased breaks in plasmid DNA in the presence of AuNPs
Brun et al.^4^	pGEM‐3Zf(−) Aqueous environment	AuNP 8 to 92 nm	5:1	49 keV effective X‐rays energy. 0–5 Gy	DEF for SSBs increased from 1.2 to 3.0 with the AuNP size increase, within a range of 8–92 nm in water.
Schlathölter et al.^15^	pBR322 4361 bp Aqueous environment	PtNP ∼3 nm	–	proton irradiation 150 MeV 0.44 keV/μm and 3.6 keV/μm 0 to 350 Gy 30 Gy/min	The presence of PtNPs not only increases the number of lesions, but also their size and lethality.
Yogo et al.^36^	pBR322 4361 bp Aqueous environment	−AuNPs and +AuNPs 1.4 nm and 30 nm	–	192Ir γ‐rays via HDR brachytherapy mean energy: 0.355 MeV Doses of 4–20 Gy	The surface charge of NPs doesn't affect the amount of generated ROS. However, +AuNPs showed DEF of 1.3 ± 0.2 for SSBs and 1.5 ± 0.4 for DSBs. While, no significant enhancement was observed in the presence of –AuNPs. The DEF for SSBs did not show a significant difference between 1.4 and 30 nm.
Xiao et al.^99^	pGEM‐3Zf(−) 3197 bp Dry films	AuNP coated with DTDTPA, S‐C11H23 and DTDTPA:Gd 5 nm, 10 and 13 nm	1:1	Electron beams 60 keV	Coatings of AuNPs may considerably attenuate short‐range low energy electrons emitted from gold, leading to a considerable decrease of radiosensitization.
Porcel et al.^100^	pBR322 4361 bp Aqueous environment	PtNPs coated with polyacrylic Acid 3 nm	2:1	C6^+^ ions 276 MeV amu−1 4Gymin−1 13.4 keV μm−1 0 up to 360 Gy	PtNPs enhances strongly the biological efficiency of radiations
Khalil et al.^71^	pUC21 3266 bp 10 ng.μL^−1^ Aqueous environment	AuNP Citrate‐coated 6, 10 and 25 nm	1:10	11, 22, 34, 45, 67, 89 Gy 1.5 keV	The SSB number per plasmid increases when, for a same mass of Au, the core size of the AuNPs decreases. The results demonstrate the damaging role of H2O2 in presence of AuNP.
Porcel et al.^49^	pBR322 4361 bp Aqueous environment	GdBNNPs 3.0 ± 1.0 nm	1:50	He2+ (energy = 150 MeV/uma) 2.33eVμm−1 4Gymin−1	The number of SSBs and DSBs increased linearly with the radiation dose
Porcel et al.^49^	pBR322 4361 bp Aqueous environment	GdBNNPs 3.0 ± 1.0 nm	1:50	C6+ (energy = 270 MeV/uma, 15613 keVμm−1, 7Gymin−1	The number of SSBs and DSBs increased linearly with the radiation dose
Porcel et al.^23^	pBR322 4361 bp Aqueous environment	PtNPs coated with polyacrylic acid 3 nm	15:1	10 Gy/min 0–250 Gy 11536 eV and 2639 eV	The number of SSBs and DSBs increased linearly with the radiation dose
Zheng et al.^98^	pGEM‐3Zf 3197 bp Aqueous environment	AuNP 5 ± 2 nm	1:1	Electron beams 1, 10 and 100 ± 05 eV	Compared to high energy electrons, LEE are at least one order of 2 magnitude larger in inducing SSB and DSB
Carter et al.^69^	Not reported 5600‐bp Aqueous environment	AuNP 2.8 ± 1.0 nm	1:10	10 keV	The enhancement increased as the concentration of radical scavenger increased.
Liehn et al.^70^	pBR322 4361 bp Aqueous environment	PtNP 2 nm	–	Co^60^ gamma source 2.2 kGy.h‐1	The number of DSB double in presence of PtNPs.

### 2.1 The DNA plasmid size and concentration effect

The effect of sample geometry and volume should be considered to ensure the reproducibility and accurate interpretation of biodosimetry measurements using an aqueous solution of DNA.^52^ Different length and concentration of DNA plasmids are employed in the aqueous solution. Giustranti et al.^53^ showed that the yield of SSB decreased with increasing DNA plasmid concentration. Ke et al.^54^ showed that the number of SSB per DNA molecule is inversely correlated with the DNA concentration. They justified their results by the fact that the mean free path of free radicals decreases by increase of DNA concentrations. The similar results was reported by Verger et al.^55^ They showed that for 5 ng/μL of plasmid, a higher percent of DNA damage was observed than for 1.25 ng/μL plasmid DNA. Using different concentration of plasmids, no significant difference in DNA damage was observed by Brabcová et al.^21^ Kong et al.^56^ indicates that the proportion between free radical damage and direct ionizing damage is independent of DNA concentration when the DNA concentration is under a certain value (50 ng/μL). It seems that the production of free radicals decreases as the concentration of DNA plasmid increases. Studies showed that, the number of SSBs per DNA molecule is linearly proportional to plasmid size.^54^, ^57^ According to Brabcová et al.,^21^ radiation‐induced plasmid DNA damage within plasmids is influenced by plasmid length. Taken together and based on their results, they confirmed that longer plasmid molecules are more radiosensitive. More studies are required to clearly investigate the effect of DNA plasmid size and concentration on its radiosensitivity and response to the irradiation.

### 2.2 The NP size effect

The NP size plays a key role in a successful NP deposition within cancer cells. Even minor changes in the characteristics of nano‐radiosensitizers as delicate systems may affect physicochemical aspects of radiosensitization, which ultimately lead to significant differences in the magnitude of the effect of NPs. Particles larger than 300 nm can limit the membrane wrapping efficiency and are potentially eliminated by macrophages, while NPs smaller than 100 nm in diameter are able to enter the tumor tissue and undergo cellular uptake.^58^ When NPs are small, only meager photon interactions occur inside the NP volume, whereas the secondary electrons resulting from these interactions mostly escape from the NP surface to reach the tumor.^26^ Large NPs suffer from a lack of escaped electrons but are able to interact more with photons.^26^ Additionally, NPs size influences ROS generation. The higher surface/volume ratio of smaller NPs leads to an increased number of active sites for NP interactions. Misawa et al.^59^ showed that ROS generation is inversely proportional to the diameter of the NP, indicating the importance of the surface/volume ratio of the NPs. Butterworth et al.^6^ indicated that a plasmid sample containing 5 and 20 nm AuNPs (gold nanoparticles) showed similar damage yields for SSBs in comparison with DSBs. In another study, Brun et al.^4^ reported that increasing the NP size within a range of 8–92 nm leads to an increase in dose enhancement factor (DEF) for SSBs from 1.2 to 3.0 for the irradiated plasmid DNA samples. Kiril et al.^38^ showed that under a certain dose rate, a more pronounced radiosensitization was detectable for smaller AuNPs. However, Yogo et al.^36^ indicated that the DEF for SSBs did not show a significant difference between 1.4 and 30 nm. This variation in results can be attributed to the difference in assessment methods and conditions (such as solution pH)

### 2.3 Plasmid‐DNA: NP molar ratio effect

Brun et al.^4^ indicated that an increase in NPs concentration in plasmid DNA samples contributes to a significant increase in supercoiled (SC) plasmid fraction. In another study, increasing DNA: AuNP ratio from 1:3 to 1:60 led to an increase in DEF from 1 to 1.4.^38^ Zheng et al.^16^ reported a DEF for SC loss of 1.5 when DNA: AuNP ratio was 1:1. In good agreement with Cho et al., ^60^ Foley and colleagues^9^ reported an enhancement factor of 2 for supercoiled DNA plasmid samples loaded with AuNPs (100:1), which irradiated with100 kVp X‐rays (for doses of 0.5–2 gray(Gy)).

### 2.4 Environment effect

The direct effects of ionizing radiation result from their immediate interaction with DNA molecules. It occurs when the ionizing radiation energy is deposited in DNA itself or transferred into the DNA after ionization of the solvation shell of DNA.^61^ In principle, the DNA solvation shell consists of ∼20–22 water molecules per nucleotide. In this context, the ionization of the DNA solvation shell produces a water radical cation, electron (radical anions), and excitations.^62^ Accordingly, the local release of a dense cluster of water radicals is very efficient to cause complex damage in the surrounding molecules. Apart from the direct DNA damage, the indirect‐type damage occurs when the ionizing radiation energy is deposited in the water surrounding the DNA (except the tightly bound water in the solvation shell), generating water radical species that can react with DNA.^61^, ^63^ Irradiating the dried form of plasmid would allow the indirect effects of radiation to be entirely suppressed, while in liquid environments, the indirect effects (such as ROS generations) are the main cause of damage.^64^ Comparison of the damage yields from aqueous and dry environments allowed the contribution of indirect effects of radiation to be measured. The indirect effect of irradiation is the dominant process in dilute solutions and has been extensively studied using a variety of aqueous model samples. To fully suppress the indirect effect of irradiation on DNA plasmid, the dried/hydrated form of plasmid can be used. Noteworthy, the presence of scavengers in liquid water, could modify the indirect effects.^65^

The electrons with energies above 20 electron volt (eV) emitted by the NPs induce water radiolysis, resulting in ROS production such as hydroxyl radicals (HO^0^). After exposure to ionizing radiation in the presence of NPs, hydroxyl radicals are likely to constitute the primary cause of DNA damage.^66^ Studies have revealed that the hydroxyl radical (OH^0^) is the main water radical responsible for SSB and DSB defects in DNA, whereas both hydrated electrons and OH^0^ induce DNA base lesions.^67^ The number of induced SSBs and DSBs depends on DNA buffer type in the presence or absence of free‐radical scavengers.^68^ Free radical scavengers inhibit the oxidation reaction and formation of ROS, or remove the ROS before they can damage vital component. Radiosensitization of high‐Z materials cannot occur in the presence of high concentrations of scavengers because even locally generated radicals are scavenged from the NPs in the vicinity of the DNA.^6^, ^69^ Porcel et al.^51^ showed that the number of SSBs and DSBs in plasmid DNA samples containing PtNPs dramatically is decreased in the presence of radical scavengers. In another study, Schlathölter et al.^15^ showed that for the proton beams with LETs of 0.44 and 3.6 keV/μm, increasing the Dimethylsulfoxide (DMSO) amount of the solution (Plasmid + PtNP) increase the scavenging capacity leading to reduction in the yields of DNA damage induced for a given dose. McMahon and colleagues^5^ reported that at higher Tris‐EDTA concentrations as a radical scavenger the number of SSBs dramatically decreased in AuNPs containing samples. While at low scavenging capacities, no significant increase in the DNA damages was observed. They concluded that a slight change in scavenger concentrations can significantly alter the overall sensitivity of plasmid DNA either by modifying its structure or altering the lifetimes and diffusion ranges of free radicals. Similar results were also reported by Carter et al.^70^ and Khalil et al.^71^ The NPs must closely associate with plasmid molecules to induce SSB and DSB directly. With these backgrounds, the hydrated or dry plasmid DNA is a suitable medium to examine the direct effect of ionizing radiation. Yokoya et al.^67^ determined yields of SSB and DSB, as well as base lesions, in X‐irradiated plasmid DNA films. They concluded that prompt strand breaks induced in hydrated DNA arise predominantly from energy deposition in the sugar moiety of DNA.

## 3 DNA ASSAYS FOR BIODOSIMETRY

About 3000 DNA lesions are produced per exposed cell with a typical therapeutic dose of around 2 Gy per fraction of ionizing radiation.^72^ Analysis and measurement of DNA DSBs and SSBs are an important biologic tools reflecting the biological effects of ionizing radiation.^73^ The conventional methods for evaluating SSBs and DSBs generation are based on changes in DNA configurations and topologies that are detected by separating different DNA topoisomerases by gel electrophoresis or liquid chromatography. Although the three forms of DNA (supercoiled, open circular, and linear) have the same mass, they differ markedly in hydrodynamic properties, making them easy to separate by Pulsed‐field electrophoresis. However, this method has low sensitivity. It is believed that conventional methods of DSB measurement in cells underestimate the true yield of DSBs.^74^ Single Molecule Force Spectroscopy (SMFS) detects DNA damage by measuring the changes in elasticity of the DNA molecule as a result of unwinding and melting of the double helix. Over conventional techniques, the SMFS method provides unique insights into how structural stability is altered under tension. Xu and colleagues visualized individual DNA lesions in irradiated plasmid DNA by AFM (atomic force microscopy) and analyzed clustered DNA damage.^75^ AFM imaging permits precise and direct determination of the distributions of SSBs and DSBs without requiring complex mathematical modeling.^54^, ^75^, ^76^, ^77^, ^78^ In another study conducted by Smiałek et al.^79^ TUNEL (terminal deoxynucleotide transferase dUTP nick end‐labeling)/ ELISA was applied to quantify the number of SSBs induced in irradiated plasmid DNA molecules. According to their findings, DSBs are more likely to be caused by two SSBs events rather than a single DSBs event. They also found that a damaged molecule is more likely to be susceptible to VUV light than an undamaged one.

## 4 LET (LINEAR ENERGY TRANSFER) OF THE RADIATION

In dosimetry, linear energy transfer (LET) is average amount of energy loss of ionizing radiation traversed per unit distance of its track. Different radiations have different LET and categorized as high and low LET radiations (Table 2). According to Monte Carlo simulations, the distribution of DNA lesions within a DNA is dependent on LET, while the average number of DNA lesions induced by a given dose is not LET dependent.^80^ The independency of DNA damage yields on LET for dry plasmids in a thin layer was confirmed by Vyšín et al.,^64^ Wyer et al.,^81^ Urushibara et al.,^82^ and Ushigome et al.^83^ Leloup et al.^74^ reported that a reduction in SSB yield is associated with high LET in aquatic environments since more SSBs participate in forming DSBs. Also, Schlathölter et al.^15^ showed that an increase in LET led to decrease in the yield of SSB from aquatic plasmid samples. However, Vyšín et al.^64^ showed that the SSB yields for liquid samples appear independent of the LET. It was proved that the clustered lesions are induced by both low‐LET^84^ and high –LET.^85^ It is known that the complexity of DNA damage affects its lethality. Indeed, a correlation has been found between the LET and the complexity of strand breaks.^80^, ^86^ In this context, the results of the study by Porcel et al.^51^ confirmed that the SSB induction decreases with increasing LET. In contrast, DSB increased with LET. It confirms that DNA damage becomes more complex as the LET of the radiation increase.^87^ It is known that for high LET radiations, the enhancement efficacy per track is more significant at the track end, while one would expect the contrary for low LET radiations.^88^ It is expected that high‐LET radiation will cause more severe and complex lesions due to its energy deposition characteristics. Accordingly, Porcel and colleagues^51^ confirmed that the complexity of DNA damage increases with LET of the radiation.

**TABLE 2:** High LET and low LET radiations

High LET radiations	Low LET radiations
Photons (X‐ray and gamma rays), Electrons	Hadrons, neutrons, Alpha particles

Care must be taken when attempting to make absolute comparisons between different conditions. According to the reviewed literature, the environment of plasmid samples could influence the LET dependence of DNA damages in the presence of NPs. Therefore, the environment of plasmids must be taken into account in the design of nano‐radiosensitization studies using different radiations with different LETs.

### 4.1 Nano‐radiosensitization of DNA plasmid under photon beams

#### 4.1.1 Energy dependence

It would be expected that indirect effects of NPs after exposure to ionizing radiation would vary as a function of energy.^5^ Indeed, radiation exposure to low LET radiation leads to indirect DNA damage, which accounts for the majority of the overall damage.^89^ Huels and colleagues suggested 10 eV as the energy threshold for DSB occurring within 10 bp in plasmid DNA.^12^ In addition, Boudaıuffa et al.^90^ demonstrated that exposing plasmid DNA [pGEM 3Zf (2), 3199 base pairs] to 3–20 eV electrons could induce substantial DNA strand breaks. Other studies provided evidence for the ability of photons as low as 7 eV to induce both SSB and DSB in irradiated DNA plasmid.^91^, ^92^ The presence of NPs could increase the production of short‐range auger electrons. Auger electrons operate within a range of 5–10 nm under physiological conditions.^9^ The efficacy of radiosensitization of NPs under photon beams significantly depends on L‐ and K‐edge binding energies of the used NP and the radiation energy and its spectral composition.^26^, ^93^ In fact, the Auger cascade production is dominant when photons with energy below the NP K or L edge energy are used. Lechtman and colleagues,^94^ showed that a higher concentration of AuNPs is required in the tumor region when photon sources are above the K‐edge of the gold nanosensitizers. The choice of optimal beam energy is an important consideration in radiotherapy. The energy dependence of NP radiosensitization under photon beams is a property of a given NP, and it is not related to the DNA type or the setup used in the experiment being used.^4^, ^5^, ^38^ The energy dependence of AuNPs nano‐radiosensitization of radiation‐induced damages has been studied by McMahon and colleagues^5^ and Brun et al.^4^ on plasmid samples. They have demonstrated that AuNPs enhance the induced damage yields, and the energy dependence of the enhancement in NP‐containing systems is determined by the physical properties of the NP against photons and not by other factors. Another study by Kiril et al.^38^ showed that an increase in the X‐ray tube voltage (40–150kev) significantly enhances the AuNPs radio‐sensitizing effect on DNA plasmid samples.

#### 4.1.2 Radiation absorbed dose dependence

The magnitude of radiation‐induced damage is proportional to the amount of absorbed photon energy. Radiation absorbed dose, reflects the amount of energy that ionizing radiation deposit in medium through which they pass. The results of the study conducted by Butterworth et al.^6^ reported roughly exponential loss of the supercoiled (SC) form of plasmids correlated with the exponential increase in open circular (OC) form and a slight increase in the linear (L) form as a function of dose in plasmid samples irradiated by 160 kVp X‐rays in the presence of AuNPs. McMahon and colleagues^5^ showed that for DNA irradiated with 25.6 keV photons in the presence of 11.9 nm AuNPs, an increase in radiation dose leads to a significant rise in OC fraction of DNA plasmids (SSB) and reduction of SC (undamaged) DNA plasmids. Also, Kiril et al.^38^ reported that 200 kVp X‐ray irradiation, the amount of the SC plasmids decrease in a dose‐dependent manner. Also, they observed increase in the C form of plasmids in the presence of AuNPs. Similar results were reported by Brun et al.^4^ They reported that the percentage decrease of the SC form was proportional to the radiation absorbed dose.

#### 4.1.3 Dose rate effect

The dose rate influence on radiosensitization cannot be explained by physical processes alone, but by physicochemical/chemical processes. The number of induced DNA damages could be influenced by dose rate changes. Presence of NPs leads to more production of free radicals. Chemical enhancement depends on the interaction of radicals with the surface of nanomaterials, and since radicals have a certain lifetime, therefore, changing the dose rate can change the chemical enhancement. While, it seems that changing the dose rate does not have any effect in the amount of physical enhancement. Small et al.^37^ and Kiril et al.^38^ indicated that in the case of DNA plasmids without NPs, DSB yield is not significantly affected by dose rate. Nevertheless, other studies have shown that changes in the dose rate could significantly affect the radiosensitization potential of metal and metal oxide NPs.^38^, ^95^, ^96^ It seems that in the presence of NPs, the number of induced DNA damages could be influenced by dose rate changes. Interestingly, this trend does not appear when the NPs are not presented.

### 4.2 Nano‐radiosensitization of DNA plasmid under electron beams

The LET from radiation to DNA is higher for electrons than x‐rays of the same energy. However, more energetic electrons may have a similar LET to the lower energy x‐ray counterpart.^97^ The low‐energy electrons (LEE) are typically known as high LET particles, meaning they can quickly lose energy in a short range. By forming transient anions that decay into the dissociative electron attachment (DEA) or autoionization channel, electrons below 15 eV can efficiently induce SSBs, DSBs, and crosslinks resulting in a dissociative state. Small et al.^37^ measured DNA damage yields in dry and aqueous environments using DNA plasmid exposed to very high energy electron beams (VHEE) of 100–200 MeV radiotherapy source. They concluded that the physical damage of DNA caused by VHEE is similar to that caused by ^60^Co X‐rays and low‐energy electrons. Zheng et al.^98^ have demonstrated the radiosensitization effect of AuNPs on plasmid DNA irradiated by electrons of a wide energy range (1, 10, and 100 eV). They concluded that DNA is chemically sensitive to LEE damage by AuNPs. In comparison to high‐energy electrons, LEEs are much more efficient in inducing SSB and DSB because of their short‐range and high cross‐section for bond fragmentation. Xiao and colleagues^99^ indicated that the formation of SSB and DSB is increased by a factor of 2.3 in the presence of AuNP. In the subsequent investigations, Zheng et al.^16^ indicated that adding AuNPs to thin films of dry plasmid DNA at a ratio of 2:1 increased plasmid damage from exposure to fast electron irradiation (60 kev) by a factor of 2.5 compared to pure DNA. They also showed that dose rate variation has no significant effect on DNA damage yields.

### 4.3 Nano‐radiosensitization of DNA plasmid under hadron irradiations

One of the propitious techniques in cancer therapy of solid tumors embedded in sensitive tissues is hadron therapy (or proton therapy and carbon therapy), where the fast atomic ions are employed as an alternative to hard x‐rays. Hadrons are three times more efficient than conventional radiations and have been used as an alternative approach in radiotherapy due to their large cross‐section of interaction with matter.^100^ In hadronic interaction, the ionization mostly occur in the outer shells of targeted atoms, leading to production of low energy electrons compared to the energy of the electrons generated as a byproduct of X‐ray interaction with matter. Moreover, when NPs are exposed to ions rather than X‐rays, they generate more low‐energy electrons. In a depth dose profile, ion beams deposit their maximum energy at the end of their tracks, known as the Bragg peak^101^ leading to more inhomogeneous dose distributions of hadrons than X‐rays. Therefore, it is necessary to apply higher concentration of NPs when irradiating plasmids with ions.^58^ DNA damages induced by NPs is amplified more strongly at the end of the particle track.^15^ The interaction of high LET ionizing radiation with DNA molecules produces localized regions with a high density of DNA damage.^102^ High LET ions interact with a high atomic number (Z) NP via Coulomb collisions. The cross‐section depends on the kinetic energy and charge of the incident particle and the impact parameter (radial distance from the particle's track to the target). Low‐energy ions can also induce severe DNA damage which is attributed to charge transfer and binary collisions.^103^ Wälzlein et al.^104^ reported the dose enhancement factor up to 2 for PtNPs irradiated by 80 MeV proton beams. It was shown by Deng et al.^105^ that the interaction of the low‐energy ions with DNA bases is sufficient to induce fragmentation in the DNA bases. By incorporating NPs into fast ions treatment, a reduced total radiation dose is delivered to the patient, reducing the radiation side effects in adjacent healthy tissues.^49^ AuNPs have been proven as promising radiosensitizers when combined with proton therapy^35^, ^106^, ^107^ due to their atomic number and excellent biological compatibility. Simulation studies^104^, ^107^ showed increased local dose distribution for AuNPs exposed by protons. Ohsawa et al.^108^ showed that the kinetics of the SSB and DSB induction in plasmid is proportional to radiation absorbed dose for 30 MeV protons. Using plasmid DNA, Usami et al.^109^, ^110^ showed that the enhancement of the SSB and the DSB in DNA irradiated by atomic ions can be observed when heavy atoms like Pt are added to DNA. Porcel et al.^51^, ^100^ reported that the combination of PtNPs with fast ions strongly enhances lethal damages in DNA, with an efficiency factor close to 2 for double‐strand breaks. Using plasmid DNA as molecular probes, Schlathölter et al.^15^ showed that the addition of 3 nm PtNPs significantly increased the number SSBs and DSBs induced by fast protons. They confirmed that the presence of PtNPs not only increases the number of lesions but also their size and lethality. In another similar study using plasmid DNA as biodosimeter, Porcel et al.^51^, ^100^ showed that the number of SSBs and DSBs increased linearly with the radiation dose in the samples irradiated with C6^+^ and He2^+^ ion beams. They confirmed that the amplification of DNA damage is more pronounced with the C6^+^ beam. Also, the linear response of absorbed dose‐SSB/DSB in the presence of NPs was reported by Schlathölter et al.^15^ They showed that the fraction of both SSB and DSB yields linearly increased by elevated radiation dose in plasmid samples containing PtNPs irradiated by proton beams (0.44 keV/μm and 3.6 keV/μm).^15^

## 5 CONCLUSION

**FIGURE 2:**
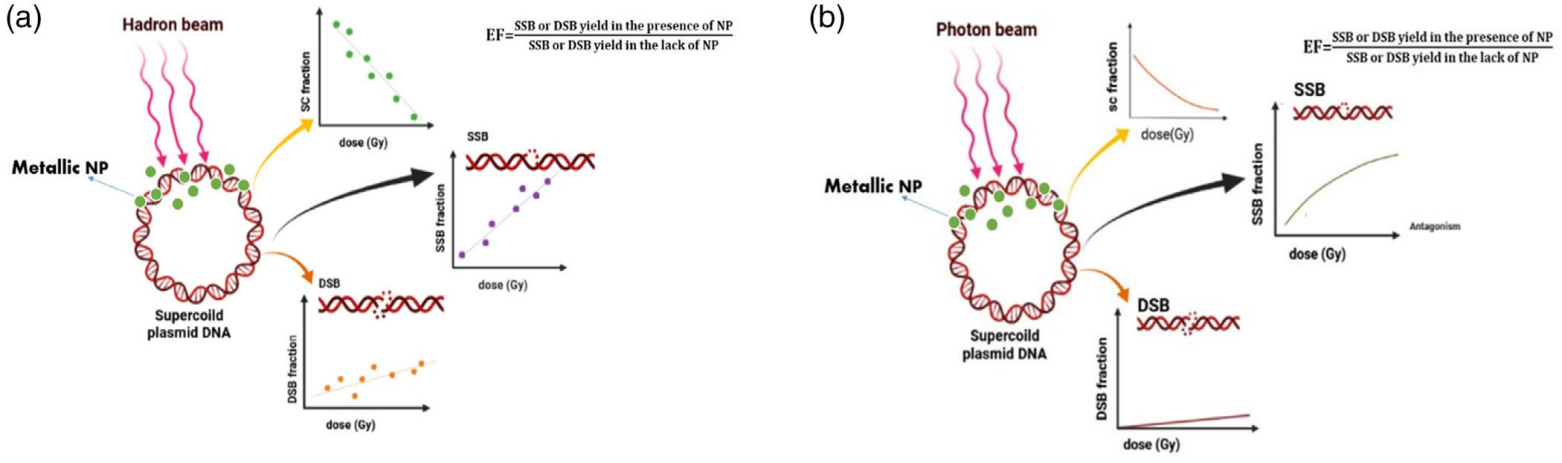
**(a)** Response function of absorbed dose‐SSB/DSB for Hadron beams. (**b)** Response function of absorbed dose‐SSB/DSB for photon beams

The DNA plasmid as a nanodosimetry probe is widely used in radiobiology and nano‐radiosensitization studies. The sensitivity of the plasmid nanodosimeter is elevated in the presence of metallic NPs and with the increase in the plasmid: NPs ratio. The different results were reported for NP size effect on radiosensitization of NPs, which could be explained by the difference in experimental conditions, such as different solutions, and irradiation conditions used in each assay. Additionally, surface properties and coating of NPs must be considered in nano‐radiosensitizer designing. It is worth emphasizing that radiations with different LETs present different behaviors against plasmids in different environments. Therefore, the environment of plasmids must be considered in the design of nano‐radiosensitization studies depending on the LET of applied radiation. For hadrons, the studies show a linear response function of absorbed dose‐SSB/DSB with implicate DSB/SSB increases with radiation dose in plasmid samples in the presence of metallic nanoparticles (Figure 2a). While for photons, an exponential relationship has been reported for dose‐response curves (Figure 2b). The influence of dose rate on the nano‐radiosensitization cannot be explained by physical processes alone and physicochemical/biochemical factors must also be considered to properly understand the mechanisms involved. The chemical dose enhancement depends on the radical interaction with the nanomaterial surface. Since the free radicals have a certain lifespan, changing the dose rate could influence chemical enhancement, while changing the dose rate does not seem to affect the rate of physical enhancement. Few studies related to the dose rate effect showed that plasmid nanodosimeters are capable of scoring dose rates in the presence of NPs. While in the samples without NPs, the dose rate effect was not seen. Based on the reviewed literature, the energy dependence of nanoparticle radiosensitization under photon beams is a property of NP and experimental setup or DNA type have no effect on it. However, there is currently no comprehensive and reliable data about the energy dependency of NP radiosensitization under hadrons and electron beams.

## AUTHOR CONTRIBUTIONS

The core idea of this study came from Elham Mansouri, Asghar Mesbahi. They also directed the other authors and analyzed the collected papers. Elham Mansouri, Asghar Mesbahi, Vahideh Tarhriz and Soheila Montazersaheb wrote the manuscript in collaboration with Tohid Ghasemnejad and Mojtaba Zarei. Final editing were done by Asghar Mesbahi and Mohammad Saied Hejazi. “All authors reviewed the manuscript.”

## CONFLICT OF INTEREST

The authors declare no conflict of interest.
